# Differential Sensitivity of Hippocampal GABAergic Neurons to Hypoxia and Ischemia-like Conditions Correlates with the Type of Calcium-Binding Protein Expressed

**DOI:** 10.3390/ijms26167966

**Published:** 2025-08-18

**Authors:** Maria V. Turovskaya, Valery P. Zinchenko

**Affiliations:** Institute of Cell Biophysics of the Russian Academy of Sciences, Federal Research Center “Pushchino Scientific Center for Biological Research of the Russian Academy of Sciences”, 142290 Pushchino, Russia; vpz@mail.ru

**Keywords:** ischemia, hypoxia, glutamate receptors, calcium-binding proteins, hypoxic preconditioning, neuronal death, calcium signaling

## Abstract

Using in vitro models of hypoxia, episodes of short-term hypoxia/reoxygenation, and ischemia-like conditions, we were able to establish different sensitivities of hippocampal neurons to their damaging factors. Using a combination of fluorescence microscopy and immunocytochemistry methods, it was possible to show that GABAergic neurons are the most vulnerable to the damaging effects of hypoxia and ischemia-like conditions, as they have a special metabolism, which is characterized by increased production of reactive oxygen species and nitric oxide. It was shown that long-term hypoxia causes the death of GABAergic neurons due to the induction of a global [Ca^2+^]_i_ increase, whereas pyramidal neurons are resistant to 30-min hypoxia. Episodes of short-term hypoxia/reoxygenation activate the phenomenon of hypoxic preconditioning in glutamatergic neurons, while this endogenous protective mechanism is absent in GABAergic neurons. Selective activation of PI3K and PKG, combined with NOS inhibition, potentiates the preconditioning effect of hypoxia/reoxygenation in glutamatergic neurons and partially activates this neuroprotective mechanism in GABAergic neurons, as indicated by diminished post-hypoxic NMDA-induced Ca^2+^ transients. These findings suggest that pharmacological intervention can protect GABAergic neuronal populations. The expression of parvalbumin, calbindin, or calretinin in the cytosol of GABAergic neurons contributes to the suppression of the global [Ca^2+^]_i_ increase during hypoxia/reoxygenation episodes, which correlates with their survival even in the absence of the hypoxic preconditioning phenomenon. Additionally, all three studied calcium-binding proteins showed potential high efficiency in maintaining the bioenergetics of GABAergic neurons during ischemia-like conditions, limiting ROS production by mitochondria and in the cytosol, and reducing nitric oxide formation. In this case, parvalbumin showed the greatest efficiency.

## 1. Introduction

Hippocampal and cortical neurons are divided into the following two main types: pyramidal neurons, which secrete glutamate, and interneurons (GABAergic neurons) with round cell bodies, which secrete γ-aminobutyric acid (GABA). On average, in the hippocampus, GABAergic neurons make up 15% of the neuronal population [1,2,3]. GABAergic interneurons are characterized by sparsely spined or aspinous dendrites, and their axons branch to create local interneuronal interactions; in addition, GABAergic neurons receive innervation from both excitatory and inhibitory synapses [4]. Accordingly, the functions of these neuronal types differ.

Pyramidal neurons are responsible for excitation, while GABAergic neurons mediate inhibition of pyramidal neurons and maintain the balance between excitation and inhibition within the network. In contrast to pyramidal neurons, GABAergic neurons are heterogeneous and are divided into subtypes based on the calcium-binding proteins (CaBPs) expressed in their cytosol. Interneurons express specific proteins, such as parvalbumin (PV), calbindin (CB), and calretinin (CR), which may promote their survival under pathological conditions [5,6].

Parvalbumin, calbindin, and calretinin have different affinities for Ca^2+^ ions in the concentration range of 10^−9^ to 10^−6^ M (Kd for CR and CB is 0.3–0.5 μM, and for PV it is 0.01–0.1 μM) [7]. CR and CB (calbindin-D28k) are fast CaBPs that can bind Ca^2+^ immediately after its cytosolic concentration increases. They share 58% structural homology and have six EF-hand domains, of which five in CR and four in CB are functional and bind Ca^2+^ ions [8]. PV has three domains, two of which can bind both Ca^2+^ and Mg^2+^; in the protein’s inactive state, these domains bind only Mg^2+^. Ca^2+^-binding by parvalbumin has slower kinetics than CR and CB and is primarily limited by the magnesium dissociation constant [9]. An important observation is that the genetic deletion of any CaBP is not compensated by increased expression of other calcium-binding proteins. In PV-knockout neurons, none of the other calcium buffers (CB-D28k, CB-D9k, or CR) are upregulated in the brain [10], leading to disruptions in intracellular signaling.

Cerebral ischemia is accompanied by the damage and death of a large number of nerve cells in in vitro and in vivo models, and some of the primary mechanisms of such damage include the excitotoxic effect of glutamate (glutamate neurotoxicity or excitotoxicity) and a global [Ca^2+^]_i_ increase due to the activation of calcium-permable glutamate receptors [11], the first isoform of Na–K–Cl cotransporter 1 (NKCC1) [12], calpains [13], and caspases [14]. Cerebral ischemia is preceded by hypoxic phenomena. The most vulnerable neurons to hypoxia and ischemia are GABAergic neurons, compared to glutamatergic neurons, and their number continuously decreases within 7 days after an hypoxic stroke [15,16]. GABAergic interneurons containing the calcium-binding protein calbindin are also damaged during hypoxia, and their death is observed within 20 days after stroke in the cerebral cortex and hippocampus [17]. The same is observed for PV-containing GABAergic neurons (within 12 days after stroke) [18]. Thus, the negative effects of prolonged hypoxia (including prenatal hypoxia) on the GABAergic component of neurotransmission persist for several weeks after an hypoxic stroke. However, this is a more prolonged cell death, whereas the absence of CaBP expression reduces the survival time of neurons. After hypoxia, not only does the number of GABAergic neurons decrease, but their location in the developing brain is also disrupted—specifically, the distance from the neuron to the nearest capillary increases compared to brains developing under normal conditions [19], a process regulated by increased levels of HIF-1α [20].

The aforementioned damaging effect of hypoxia is mediated by long-term hypoxic episodes. At the same time, positive effects of short-term hypoxic episodes on brain tissue have been established, where the phenomenon of hypoxic preconditioning is activated. Hypoxic preconditioning is achieved through short-term and repeated induction of hypoxic conditions that increase resistance to lethal hypoxia or global ischemia in various organs [21]. There are no pre-existing mechanisms in the body for such adaptation to long-term hypoxia, but there are genetically determined prerequisites that are realized under certain conditions. Under conditions of oxygen deficiency, various protective mechanisms are activated in brain cells that help prevent irreversible disruptions in the functioning of tissues and organs. These include increased activity of stress-limiting systems and oxygen transport and ATP resynthesis systems; acceleration of the conversion of short-term memory into long-term memory; increased RNA and protein synthesis in brain cells; activation of the cytochrome P450 detoxification system and the antioxidant system; reduced levels of immune complexes in the blood; and exertion of antiallergic and antineoplastic effects [22]. Thus, activation of the endogenous protective mechanism of neurons in the form of hypoxic preconditioning is a complex process and has been poorly studied to date.

This research aims to investigate the mechanisms underlying the selective vulnerability of hippocampal GABAergic neurons to hypoxia and ischemia, focusing on the role of calcium-binding proteins (PV, CB, and CR) in regulating intracellular Ca^2+^ homeostasis, suppressing oxidative stress, and activating neuroprotective pathways (PI3K, PKG, and NOS) while also evaluating the potential for pharmacological modulation to activate the hypoxic preconditioning phenomenon in GABAergic neurons.

## 2. Results

### 2.1. Differential Effects of Prolonged Hypoxia and Episodes of Short-Term Hypoxia/Reoxygenation on Ca^2+^ Signals of Hippocampal Neurons in Culture

**Figure 1:**
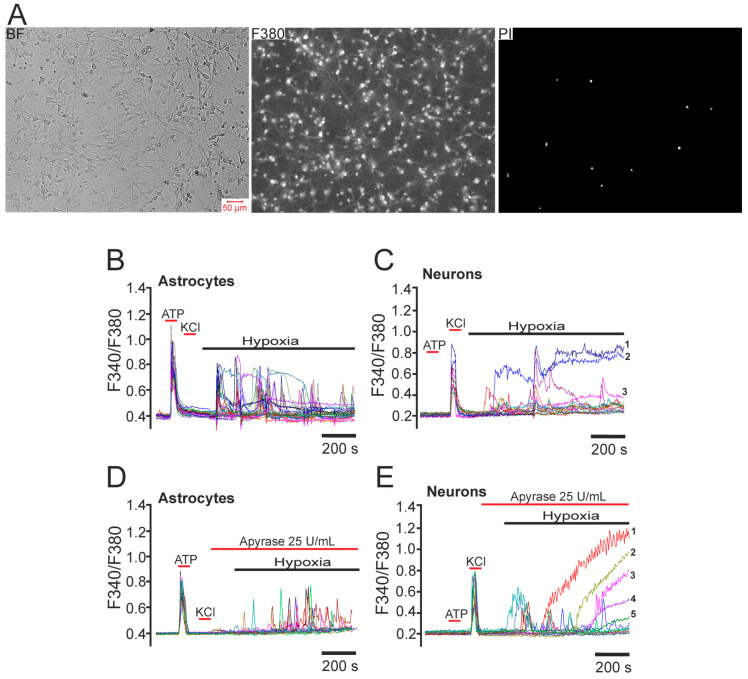
The effect of prolonged hypoxia (~30 min) on astrocytes and neurons of the hippocampus in primary cell culture: (**A**) images of rat hippocampal cell culture on the 10th day in vitro (DIV) in transmitted light (BF), fluorescence of the calcium-sensitive probe Fura-2 in the 380 nm registration channel (F380), and fluorescence of nuclei stained with propidium iodide (PI) after hypoxia; (**B**,**C**) Ca^2+^ signals of hippocampal astrocytes (**B**) and neurons (**C**) under hypoxic conditions; (**D**,**E**) Ca^2+^ signals of hippocampal astrocytes (**D**) and neurons (**E**) under hypoxic conditions after application of the ATP-cleaving enzyme apyrase (25 U/mL). ATP and KCl—application of 10 μM ATP and 35 mM KCl to identify astrocytes (characterized by Ca^2+^ signals in response to ATP) and neurons (characterized by Ca^2+^ signals in response to KCl). Labels 1–5: neurons with a global [Ca^2+^]_i_ increase. For each panel, the experiments were performed in seven replicates.

To identify neurons, the experiment included a short-term (30 s) test application of 35 mM KCl. The KCl-induced depolarization promotes the opening of voltage-gated Ca^2+^ channels and induces rapid Ca^2+^ responses exclusively in neurons. The conductivity and density of cation channels in astrocytes are insufficient to evoke a high-amplitude Ca^2+^ response to KCl. However, application of 10 µM ATP induced activation of purinoreceptors and Ca^2+^ responses exclusively in astrocytes [23,24,25]. In the primary mixed-neuroglial culture of rat hippocampus (10 DIV, Figure 1A) stained with the calcium-sensitive probe Fura-2 AM, a gradual decrease in pO_2_ (to 23 ± 15 mmHg) caused Ca^2+^ signals of various shapes in 32 ± 18% of astrocytes (Figure 1B) and 15 ± 9% of neurons (Figure 1C). The pre-hypoxic pO_2_ in the HBSS media was 97 ± 11 mmHg. Moreover, on average, the number of astrocytes responding with a rapid [Ca^2+^]_i_ increase was significantly greater than that of neurons, and their Ca^2+^ responses most often appeared as high-amplitude Ca^2+^ oscillations, whereas short-term signals were characteristic of neurons. For single hippocampal neurons (Figure 1C, marked 1–3), a global (irreversible over the entire recording interval) [Ca^2+^]_i_ increase was detected. This global [Ca^2+^]_i_ increase is an indicator of cell damage, since staining of cells in the microscope’s field of view after recording hypoxia-induced Ca^2+^ dynamics using propidium iodide showed necrotic death of single cells (Figure 1A—PI). It is known that astrocytes are capable of contributing to neuronal signaling [26,27]. Application of the ATP-cleaving enzyme apyrase before hypoxia induction resulted in a decrease in hypoxia-induced Ca^2+^ activity in astrocytes (Figure 1D), whereas the same trends persisted in neurons: hypoxia-induced transient Ca^2+^ signals and a global [Ca^2+^]_i_ increase were recorded in individual neurons (Figure 1E).

In the primary mixed-neuroglial culture of rat hippocampus (10 DIV, Figure 1A), prolonged hypoxia induced Ca^2+^ signals of different shapes in astrocytes (Figure 1B) and neurons (Figure 1C). In astrocytes, the rapid increase in [Ca^2+^]_i_ most often took the form of high-amplitude oscillations, whereas short-term signals were characteristic of neurons. For single hippocampal neurons (Figure 1C, marked 1–3), a global (irreversible over the entire recording interval) increase in [Ca^2+^]_i_ was detected. This global [Ca^2+^]_i_ increase is an indicator of cell damage and death, as confirmed by necrotic nuclei stained with propidium iodide (Figure 1E). Although calcium imaging revealed a global increase in [Ca^2+^]_i_ in GABAergic neurons during hypoxia, direct assessment of cell viability using propidium iodide staining confirmed necrotic death in these populations (Figure 1A,E). Thus, the observed [Ca^2+^]_i_ disturbances correlate with cell death but do not provide direct evidence of this process.

**Figure 2:**
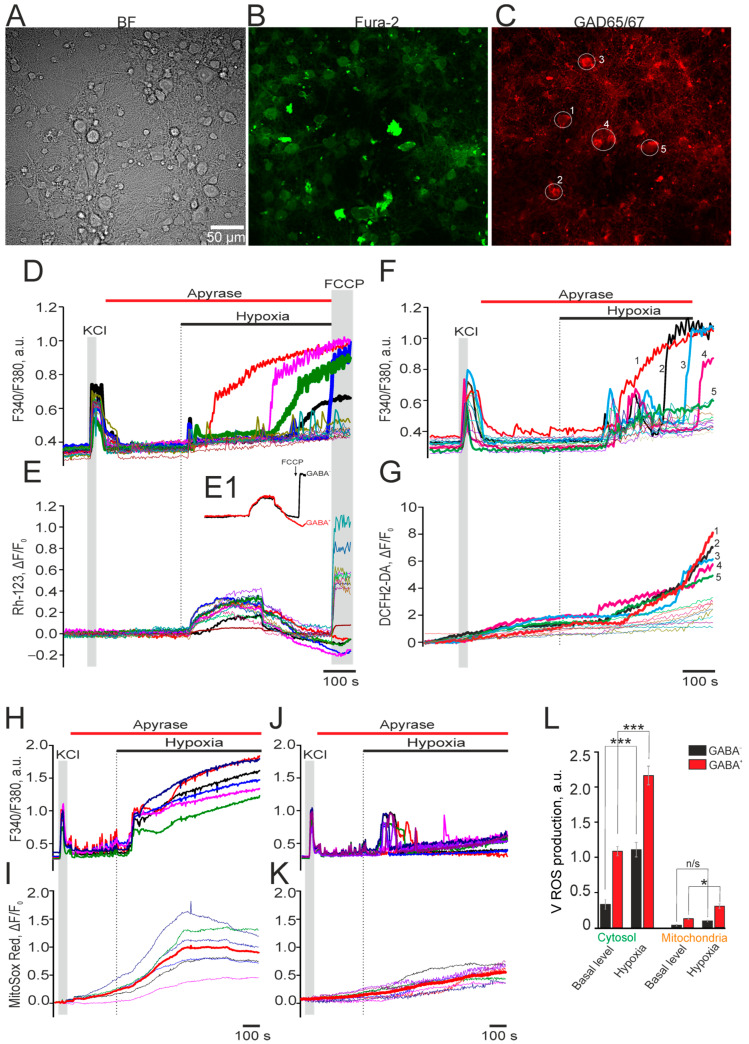
Differential effects of prolonged hypoxia on Ca^2+^ signaling and ROS production in hippocampal GAD 65/67(-) and GAD 65/67(+) neurons. (**A**–**C**) Transmitted microscopy images (**A**) of hippocampal cell culture corresponding to Fura-2 images (380 nm channel) (**B**) and immunocytochemical staining with antibodies against glutamate decarboxylase GAD65/67 (**C**). The white circles and numbers in panel (**C)** indicate GAD 65/67(+) neurons. (**D**,**E**) [Ca^2+^]_i_ dynamics (**D**) and mitochondrial membrane potential (**E**) in GAD 65/67(-) and GAD 65/67(+) (numbers 1–5) neurons during hypoxia. (**E1**) Averaged values of mitochondrial membrane potential of GAD 65/67(-) (black curve) and GAD 65/67(-) (red curve) neurons during hypoxia and in response to the application of 1 μM of a mitochondrial respiratory chain uncoupler—FCCP (to detect mitochondria functionality). (**F**,**G**) Simultaneous [Ca^2+^]_i_ dynamics registration (**F**) and ROS production (**G**) in the cytosol of GAD 65/67(-) and GAD 65/67(+) (numbers 1–5) neurons during hypoxia. Numbers indicate signals of GAD 65/67(+) neurons and correspond to the images in panels (**C**,**H**,**I**). Simultaneous [Ca^2+^]_i_ dynamics (**H**) and ROS production (**I**) by mitochondria of GAD 65/67(+) neurons during hypoxia. (**J**,**K**) Simultaneous [Ca^2+^]_i_ dynamics registration (**J**) and ROS production (**K**) by mitochondria of GAD 65/67(-) neurons during hypoxia. (**L**) Rate of ROS production in the cytosol and mitochondria of GAD 65/67(-) (black bars) and GAD 65/67(+) (red bars) neurons during hypoxia. Averaged data for 63 (GAD 65/67(+) neurons) and 620 (GAD 65/67(-) neurons) cells are presented. Hypoxia was induced in the presence of the ATP-degrading enzyme apyrase (25 U/mL) to exclude the contribution of ATP secretion by astrocytes. KCl—short-term depolarization using a 35 mM KCl application to detect neurons. Statistical analyses were performed with the Student’s *t*-test. Differences are significant at *** *p* < 0.001 and * *p* < 0.05; n/s—differences are not significant (*p* > 0.05). For each panel, experiments were performed in 5 replicates.

In hippocampal cell culture under our experimental conditions, GABAergic neurons account for approximately 5–20% of all neurons, which is consistent with the in vivo ratio [28]. Therefore, we hypothesized that the significant differences in neuronal Ca^2+^ dynamics, as shown in Figure 1, are associated with the differential effects of hypoxia on GABAergic and glutamatergic neurons. Neuroimaging was used to simultaneously record the following two parameters: [Ca^2+^]_i_ dynamics as a reference channel for identifying the cellular response to hypoxia and, as a parameter of cellular physiology, mitochondrial membrane potential, as well as ROS production in the cytosol or mitochondria. After the experiments, the cells were fixed and stained with antibodies against glutamate decarboxylase (GAD65/67) to identify GABAergic neurons. Antibodies against GAD65/67 serve as standard tools for identifying GABAergic neurons, as these glutamate decarboxylase isoforms are essential for GABA synthesis. Studies demonstrate that GAD67 is expressed in 90–95% of GABAergic neurons in the CNS [29]. To exclude even minimal contributions from hypoxia-induced astrocytic Ca^2+^ signals and to facilitate neuronal detection, the experimental series shown in Figure 2 was performed with the addition of the ATP-cleaving enzyme apyrase (25 U/mL).

We hypothesized that the significant differences in Ca^2+^ dynamics observed in neurons (Figure 1) reflect the differential effects of hypoxia on GABAergic and glutamatergic neurons, which were identified using antibodies against glutamate decarboxylase (GAD65/67). Astrocytic Ca^2+^ signals during the experiments (Figure 2) were suppressed by adding the ATP-cleaving enzyme apyrase (25 U/mL).

The recording of neuronal Ca^2+^ dynamics (Figure 2A) in cells loaded with the Ca^2+^-sensitive probe Fura-2 AM (Figure 2B), followed by GAD65/67 immunostaining (Figure 2C), revealed that during hypoxia, hippocampal neurons exhibited either single Ca^2+^ transients or (less frequently) Ca^2+^ oscillations with variable lag periods (Figure 2D). In most GABAergic (GAD65/67(+)) neurons, 8–10 min of hypoxia induced a global (irreversible) [Ca^2+^]_i_ increase (Figure 2D, thick lines), leading to cell death, as confirmed by their lack of response to FCCP (Figure 2E, thick lines). A small subset of GAD 65/67 (+) neurons showed no hypoxic Ca^2+^ responses (not shown).

Mitochondrial membrane potential analysis showed that rhodamine-123 fluorescence increased by 40 ± 25 s after hypoxia onset in both GAD65/67(−) and GAD65/67(+) neurons, indicating hypoxia-induced depolarization. However, while GAD65/67(−) neurons recovered their resting potential (Figure 2E,E1, black curve), GAD 65/67(+) neurons showed further depolarization (Figure 2E,E1, red curve). Application of 1 μM FCCP (a mitochondrial uncoupler) failed to induce Rh-123 fluorescence increases in GAD 65/67 (+) neurons, unlike in GAD65/67(−) neurons. This observation, combined with the global [Ca^2+^]_i_ increase, confirms that GAD 65/67(+) neurons are damaged during hypoxia (Figure 2E1).

One of the sources of cellular ROS production may be enzymes localized in the cytosol. Measurement of ROS production in the cytosol of hippocampal neurons using the H_2_DCF-DA probe shows that during hypoxia in GABA^+^ neurons (labeled 1–5), a global [Ca^2+^]_i_ increase occurs (Figure 2F) and coincides in time with the phase of rapid ROS production (Figure 2G, cells labeled 1–5), whereas in GABA^−^ neurons similar effects of hypoxia were not recorded, despite the presence of various types of Ca^2+^ signals (Figure 2F, thin curves). The majority of ROS in neurons is produced by mitochondria [30]. In GABA^+^ neurons, during the hypoxia-induced global [Ca^2+^]_i_ increase (Figure 2H), there was an increase in ROS production by mitochondria (Figure 2I). Whereas in GABA^−^ neurons, a predominantly transient [Ca^2+^]_i_ increase was recorded (Figure 2J), which did not correlate with the increase in ROS production during hypoxia (Figure 2K). Analysis of the ROS production rates in GABA^+^ and GABA^−^ neurons showed that both GABA^−^ and GABA^+^ neurons were characterized by basal (pre-hypoxic) ROS production in the cytosol, the rate of which is, on average, 80% higher in GABA^+^ neurons (Figure 2L) compared to GABA^−^ neurons. Hypoxia induced a cytosolic ROS production increase in both types of neurons, but in GABA^+^ neurons the ROS production rate induced by hypoxia was 110% higher compared to GABA^−^ neurons (Figure 2L). Our experiments show that basal ROS production in the mitochondria of GABA^+^ neurons was 145% higher (Figure 2L) than in GABA^−^ neurons (Figure 2L). During hypoxia, there was no significant increase in the ROS production rate in the mitochondria of GABA^+^ neurons (Figure 2L), despite the presence of transient Ca^2+^ signals (Figure 2J). At the same time, in GABA^+^ neurons, an ROS production increase of 35% compared to the resting level was observed (Figure 2L).

Thus, neurons and astrocytes can sense decreasing pO_2_ levels in their environment and respond by generating Ca^2+^ signals. Astrocytes from different brain regions exhibit high homogeneity in Ca^2+^ signaling during hypoxia without developing a global [Ca^2+^]_i_ increase, whereas neurons show significant response heterogeneity, including global [Ca^2+^]_i_ increases specifically in GABAergic (GAD65/67(+)) neurons. Notably, GAD65/67(+) neurons demonstrate greater hypoxia sensitivity than glutamatergic neurons, manifested by elevated ROS production in both cytosol and mitochondria during hypoxia. This differential response may be a key determinant of intracellular signaling disruptions and the selective vulnerability of certain GAD65/67(+) neuron populations.

**Figure 3:**
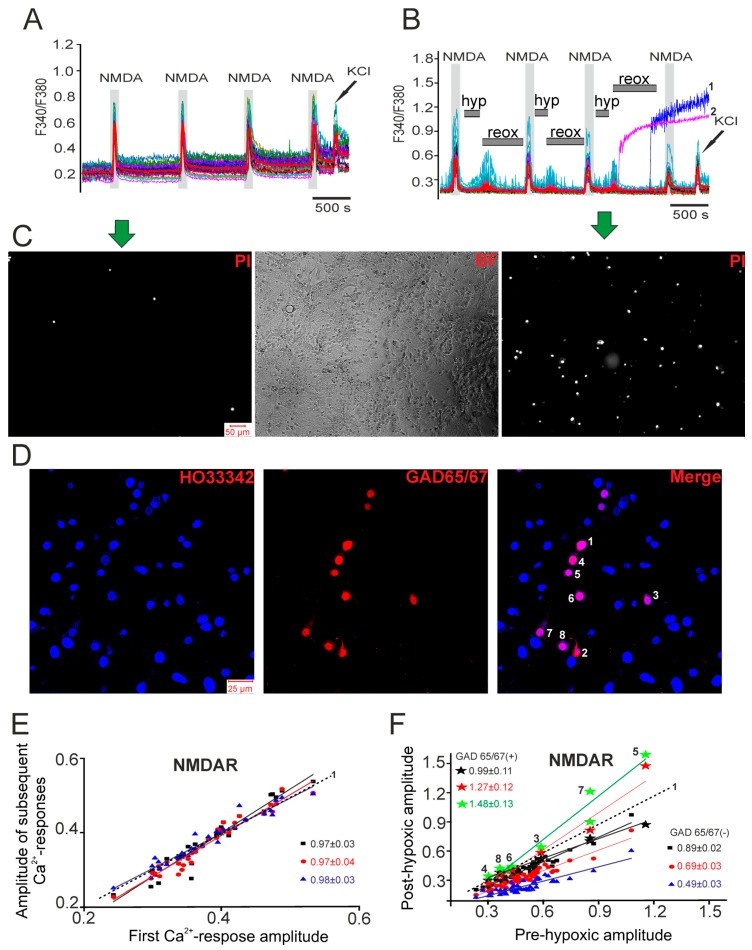
Effects of short-term hypoxia and reoxygenation episodes on hippocampal neurons. (**A**) Applications of 10 μM NMDA receptor activator (N-methyl-D-aspartate, NMDA) in a magnesium-free medium without short-term hypoxia episodes. (**B**) Applications of 10 μM NMDA in a magnesium-free medium before (first NMDA application) and after short-term hypoxia (hyp, 3 min) and reoxygenation (reox, ~10 min) episodes. (**C**) Staining of hippocampal cells with propidium iodide after NMDA applications without hypoxia episodes and in a model with repeated hypoxia/reoxygenation episodes. BF—transmitted light image of the cell culture. (**D**) Staining of hippocampal cells, the Ca^2+^ signals of which are shown in panel (**B**) with antibodies against glutamate decarboxylase (GAD65/67). HO33342—cell nuclei are stained with Hoechst 33342. Merge—HO33342 fluorescence and secondary antibodies against GAD65/67. (**E**) Linear approximations of the Ca^2+^ responses of hippocampal neurons to repeated applications of 10 μM NMDA in a magnesium-free medium. (**F**) Linear approximations of the dependences of the Ca^2+^-response amplitudes to NMDA applications in the control (1), after the first (black straight lines), second (red straight lines), and third (blue straight lines) episodes of hypoxia in GAD65/67(−) and GAD65/67(+) (indicated by numbers 3–8) neurons. For each panel, the experiments were performed in 7 replicates.

Hypoxia exhibits both detrimental and protective effects. Short-term hypoxia episodes protect neurons from subsequent ischemic damage by activating preconditioning mechanisms that reduce [Ca^2+^]_i_ overload [31,32]. Using Fura-2-loaded hippocampal neurons, we recorded [Ca^2+^]_i_ changes during NMDA receptor (NMDAR) activation before and after hypoxia–reoxygenation (H/R) episodes. Brief NMDA application (10 μM, Mg^2+^-free medium) evoked consistent [Ca^2+^]_i_ responses (Figure 3A,B). After three H/R cycles (3 min hypoxia/10 min reoxygenation), response amplitudes progressively decreased, from 0.35 to 0.06 (Figure 3B, red curve), while reoxygenation triggered synchronous [Ca^2+^]_i_ transients (Figure 3B), reflecting network hyperexcitability. Two neurons showed global [Ca^2+^]_i_ increases during the third reoxygenation, indicating damage. H/R increased neuronal death (18–20% vs. <1% in the controls; Figure 3C). Immunostaining revealed that global [Ca^2+^]_i_ increases occurred exclusively in GAD65/67(+) neurons (Figure 3D). While most neurons showed reduced NMDAR responses post-H/R (slope decrease: 0.89 ± 0.02 to 0.49 ± 0.03; Figure 3F), surviving GAD65/67(+) neurons exhibited enhanced responses (slope increase: 0.99 ± 0.11 to 1.48 ± 0.13; Figure 3F). This dichotomy suggests GABAergic-neuron-specific plasticity during H/R.

Thus, in hippocampal glutamatergic neurons, episodes of short-term hypoxia cause suppression of the amplitude of Ca^2+^ signals upon application of NMDA receptor activator, which indicates the development of the hypoxic preconditioning phenomenon. In GAD65/67(+) neurons, hypoxic preconditioning was not activated, but, on the contrary, a toxic effect occurred, the manifestation of which was an increase in the amplitude of Ca^2+^ signals upon NMDAR activation after episodes of hypoxia/reoxygenation, or we observed the damage and death of some GAD65/67(+) neurons during reoxygenation periods. In addition, in a number of experiments associated with NMDAR activation, hypoxia–reoxygenation episodes caused the appearance of symptoms of post-hypoxic hyperexcitation in glutamatergic and GAD65/67(+) neurons during reoxygenation, correlating with the generation of a global [Ca^2+^]_i_ increase in some GAD65/67(+) neurons.

**Figure 4:**
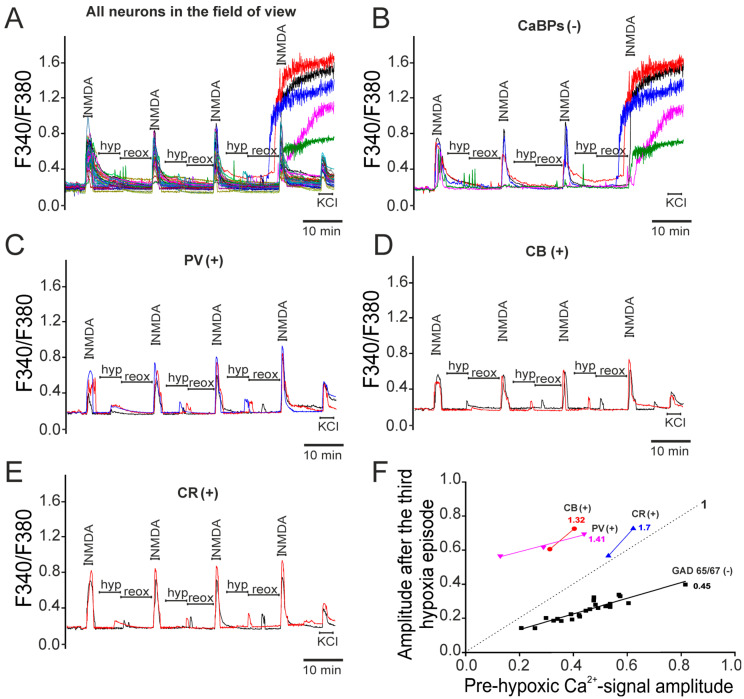
Effects of short-term hypoxia/reoxygenation episodes on Ca^2+^ signals during repeated applications of 10 μM NMDA in magnesium-free medium in different subtypes of GABAergic neurons depending on the expressed calcium-binding protein. (**A**) Ca^2+^ signals of all neurons in the field of view of the microscope during applications of 10 μM NMDA in magnesium-free medium, episodes of short-term hypoxia (hyp) and reoxygenation (reox, ~10 min). (**B**–**E**) Ca^2+^ signals during NMDA applications and short-term hypoxia/reoxygenation episodes in GABAergic neurons (GAD 65/67 (+)) without expression of the studied calcium-binding proteins ((**B**) CaBPs (-)), expressing parvalbumin ((**C**), PV (+)), calbindin ((**D**), CB (+)), or calretinin ((**E**), CR (+)). (**F**) Dependences of the amplitudes of the Ca^2+^ response to NMDA after episodes of short-term hypoxia (relative units) on the amplitudes of the Ca^2+^ response before hypoxia (relative units) depending on the presence of CaBPs in GAD 65/67 (+) neurons. The amplitudes of the Ca^2+^ responses and their approximations by a linear function of GAD 65/67 (+) neurons (black squares), GAD 65/67 (+) neurons that express calretinin (CR (+), blue straight line), calbindin (CB (+), red straight line), or parvalbumin (PV (+), pink straight line) are shown. Straight line 1 is a linear approximation of the data from the control (without hypoxia). For each panel, the experiments were performed in 4 replicates.

To investigate the role of Ca^2+^-binding proteins in the functioning of GAD65/67(+) neurons during hypoxia (Figure 4A), after the Ca^2+^-signals’ registration, the cells were stained with antibodies against GAD65/67. The Ca^2+^ signals of hippocampal GAD65/67(+) neurons that did not contain the studied calcium-binding proteins are plotted separately in Figure 4B, where it can be seen that all neurons recorded a global [Ca^2+^]_i_ increase during reoxygenation, whereas the GAD65/67(+) neurons that expressed parvalbumin (Figure 4C), calbindin (Figure 4D), or calretinin (Figure 4E) survived the hypoxia/reoxygenation episode model. An analysis of the amplitudes of the Ca^2+^ signals during NMDAR activation after three episodes of hypoxia/reoxygenation showed that in glutamatergic neurons (GAD65/67(−)) there was a decrease in the amplitudes of the Ca^2+^ signals and the slope of the linear approximation of the amplitudes was 0.45 (Figure 4F—GAD65/67(−)), which indicates a clearly expressed phenomenon of hypoxic preconditioning. The slope of the linear functions approximating the amplitudes of the Ca^2+^ signals of GAD65/67(+) neurons expressing parvalbumin, calbindin, or calretinin were 1.41, 1.32, and 1.7, respectively (Figure 4F). That is, in GAD65/67(+) neurons expressing calcium-binding proteins, the phenomenon of hypoxic preconditioning was not activated, but, on the contrary, the episodes of hypoxia/reoxygenation caused toxic effects that contributed to an increase in the amplitudes of the Ca^2+^ signals during NMDAR activation.

Thus, the expression of calcium-binding proteins in the cytosol of GABAergic neurons is associated with their survival during hypoxia/reoxygenation episodes. However, residual adverse effects persist, manifested by the absence of hypoxic preconditioning and an increase in Ca^2+^-signal amplitudes upon NMDA exposure after three hypoxia/reoxygenation cycles.

To investigate the intracellular mechanisms underlying hypoxic preconditioning activation, we conducted a pharmacological modulation analysis. In our short-term hypoxia/reoxygenation model, we added signaling protein inhibitors/activators after the first hypoxic episode. Subsequent episodes assessed the preconditioning efficacy through NMDA-induced Ca^2+^-signal amplitudes (Table 1). PI3K inhibitors (LY-294002, wortmannin) dose-dependently suppressed preconditioning in glutamatergic neurons and exacerbated toxic effects in GAD65/67(+) neurons, increasing their mortality. Conversely, the PI3K activator 740Y-P enhanced preconditioning in glutamatergic neurons (slope coefficient: 0.36 ± 0.06 vs. the control’s 0.47 ± 0.07) and induced it in GAD65/67(+) neurons (0.63 ± 0.04 vs. 1.12 ± 0.05) while reducing neuronal death to 1%. Since PI3K/Akt activates NO synthase, we examined NO’s dual role. The NOS inhibitor 7NI maintained preconditioning (glutamatergic: 0.46 ± 0.07; GABAergic: 0.56 ± 0.21), while the NO donor SNAP abolished it, increasing cell death to 16%. Notably, L-arginine (NOS substrate/α2-adrenergic activator) promoted preconditioning (glutamatergic: 0.48 ± 0.07; GABAergic: 0.54 ± 0.2), likely by generating moderate, signaling-competent NO levels versus SNAP’s toxic excess. PKG inhibition (Rp-8-pCPT-cGMPS) suppressed preconditioning, increasing GAD65/67(+) neuron death to 11%. The cGMP/PKG activator 8-Br-cGMP preserved glutamatergic neuron preconditioning (0.49 ± 0.06 vs. the control’s 0.47 ± 0.07) and activated it in GABAergic neurons (0.8 ± 0.07 vs. 1.12 ± 0.05), confirming PKG’s protective role via IP3R inhibition and Ca^2+^ homeostasis regulation.

Thus, the results of the pharmacological modulation analysis demonstrate that PI3K, PKG, and NO synthase are involved in the mechanism of rapid hypoxic preconditioning in both glutamatergic (GAD65/67(−)) and GAD65/67(+) neurons. Activation of these pathways promotes preconditioning (particularly in GABAergic neurons) and, consequently, reduces the death of GABAergic neurons that are most vulnerable to hypoxia.

### 2.2. Expression of Calcium-Binding Proteins Correlates with GABAergic Neuron Survival and Reduced Oxidative Stress Under Ischemia-like Conditions

**Figure 5:**
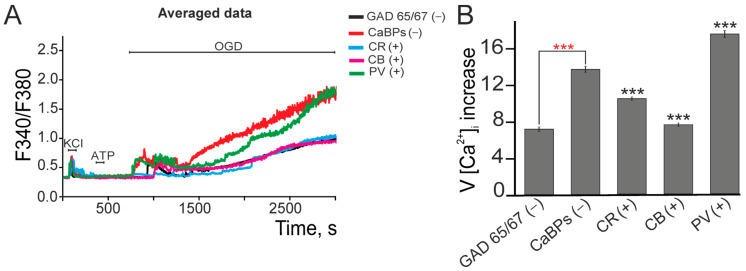
Ca^2+^ signals in different types of hippocampal neurons under ischemia-like conditions (OGD, ~40 min) and the role of calcium-binding protein expression in GABAergic (GAD65/67(+)) neurons. (**A**) Averaged Ca^2+^ signals of GAD65/67(+) neurons, GAD65/67(+) neurons without expression of the studied calcium-binding proteins (CaBPs(-)), GAD65/67(+) neurons expressing calretinin (CR(+)), calbindin (CB(+)), or parvalbumin (PV(+)). (**B**) Effect of CaBP expression in GAD65/67(+) neurons on the rate of OGD-induced global [Ca^2+^]_i_ increase. To measure the rate of global [Ca^2+^]_i_ increase, Ca^2+^ signals from different neuron types were averaged (shown in panel **A**). The rate of [Ca^2+^]_i_ increase was estimated from the averaged Ca^2+^ signals from the onset of the global [Ca^2+^]_i_ increase to the end of the experiment. Statistical analyses were performed using the Student’s *t*-test. Differences are significant at *** *p* < 0.001. Black asterisks indicate comparisons between the experimental groups and the CaBPs(-) group. Red asterisks indicate a comparison between the GAD65/67(−) group and the CaBPs(-) group. The experiments were performed in 6 replicates for each panel. Individual Ca^2+^ signals are shown in Supplementary Figure S1.

Previous studies have demonstrated that 40 min of oxygen–glucose deprivation (OGD) induces death in 80 ± 18% of cells [33]. Our analysis of calcium dynamics reveals that neurons typically exhibit biphasic [Ca^2+^]_i_ elevations during OGD—an initial reversible phase followed by irreversible accumulation (Figure 5A), though with population-specific variations in steady-state levels. Immunohistochemical characterization showed that GAD 65/67 (-)- neurons displayed initial [Ca^2+^]_i_ transients with subsequent recovery before a delayed global elevation (7.7 a.u.) (Figure 5B), while CaBP-negative GAD 65/67(+)- neurons showed immediate sustained elevations (14.5 a.u.) without a recovery phase (Figure 5A,B). Parvalbumin-expressing GAD65/67(+) neurons maintained both phases but with elevated [Ca^2+^]_i_ accumulation rates compared to their CaBP-negative counterparts. In contrast, calbindin- or calretinin-positive Gad 65/67 (+) neurons exhibited attenuated global [Ca^2+^]_i_ increases, with CR+ neurons additionally suppressing reversible phase transients. Calcium imaging revealed that GABAergic neurons (GAD65/67(+)) exhibited a robust increase in [Ca^2+^]_i_ upon OGD (Figure 5A), which, in previous experiments (Figure 1), correlated with the loss of viability. In contrast, neurons expressing calcium-binding proteins (CB or CR) exhibited significantly smaller changes in the Ca^2+^ responses (Figure 5B), which may indicate their increased resistance to ischemic conditions. Notably, CB/CR expression was associated with lower OGD-induced [Ca^2+^]_i_ accumulation in GAD65/67(+) neurons, reaching levels similar to those in GAD65/67-negative populations (Figure 5A,B). This correlation suggests a potential link between calcium-binding proteins and hypoxic susceptibility, possibly involving differential regulation of calcium homeostasis.

**Figure 6:**
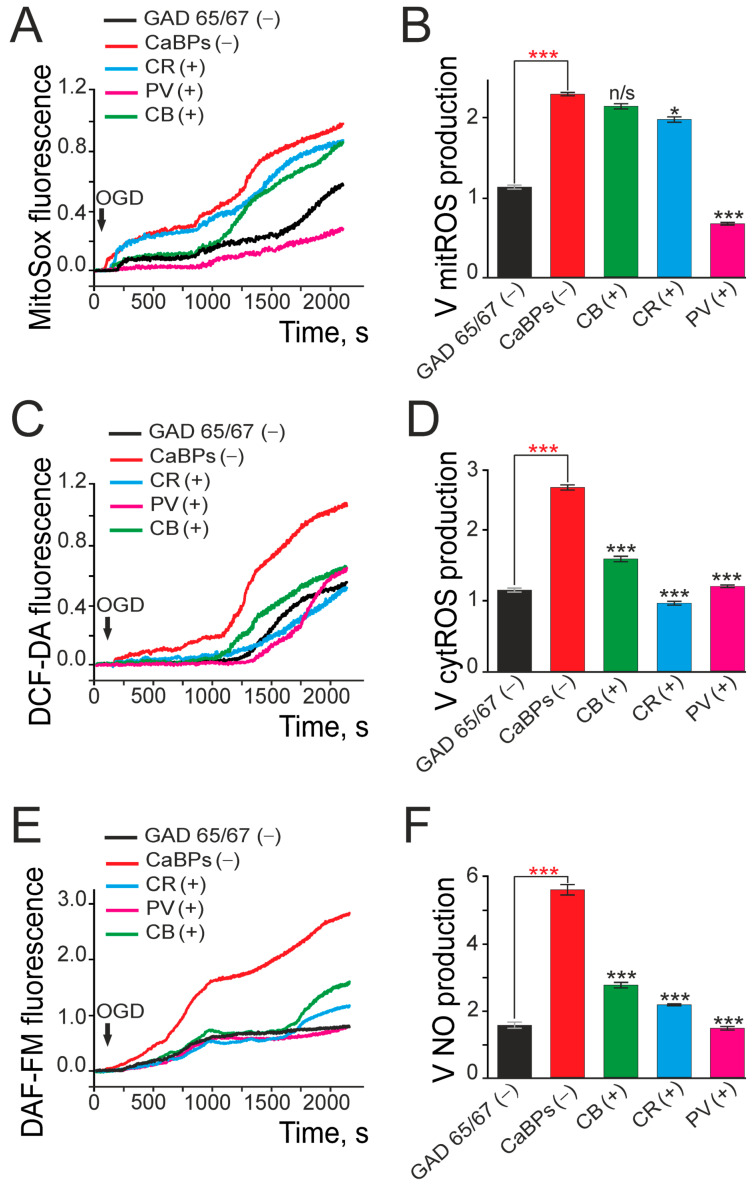
Mitochondrial ROS production (**A**), cytosolic enzyme-derived ROS (**C**), and nitric oxide formation (**E**) in GAD65/67(+) or GAD65/67(−) neurons under OGD conditions, analyzed by calcium-binding protein expression. Panels (**A**,**C**,**E**) show the averaged curves (10 cells per curve) of mitochondrial ROS production (**A**), cytosolic ROS (**C**), and nitric oxide production (**E**) in GAD65/67(−) or GAD65/67(+) neurons. Panels (**B**,**D**,**F**) display the rates of mitochondrial ROS production (**B**), cytosolic ROS (**D**), and nitric oxide production (**F**). Statistical analyses were performed using the Student’s *t*-test. Significant differences are indicated at * *p* < 0.05 and *** *p* < 0.001; n/s—not significant (*p* > 0.05). Black asterisks denote comparisons between the experimental groups expressing CaBPs and the CaBPs(−) control group. Red asterisks indicate comparisons between GAD65/67(−) neurons and the CaBPs(−) group. The experiments were performed in quadruplicate for each panel.

Free radical generation during ischemia is a key contributor to brain cell death [34]. Measurement of mitochondrial ROS production during OGD in different types of hippocampal neurons showed that CaBPs(-) GAD65/67(+) neurons had a more than 2-fold higher mitochondrial ROS production rate compared to CaBPs(-) GAD65/67(−) (glutamatergic) neurons (Figure 6A,B). Expression of calbindin or calretinin in GAD65/67(+) neurons showed no association with changes in ROS production, whereas parvalbumin expression was correlated with significantly lower ROS levels in these neurons (Figure 6A,B). Moreover, cytosolic ROS measurements revealed that the expression of any of the three studied CaBPs was correlated with reduced ROS production by cytosolic enzymes in GAD65/67(+) neurons during OGD (Figure 6C,D). Nitric oxide (NO), which is also actively produced during cerebral ischemia [35], showed a corresponding modulation: expression of all three CaBPs was associated with lower NO production in GAD65/67(+) neurons under OGD conditions, with parvalbumin showing the strongest inverse correlation (Figure 6E,F).

Thus, the expression of CaBPs in the cytosol of GAD65/67(+) neurons was associated with a reduced sensitivity to ischemia-like conditions (oxygen-glucose deprivation, OGD), corresponding to lower global [Ca^2+^]_i_ increases and decreased production of free radicals. Interestingly, while parvalbumin expression showed no correlation with the rate of [Ca^2+^]_i_ increase during OGD, it was uniquely associated among the studied calcium-binding proteins with reduced ROS levels in the mitochondria of GAD65/67(+) neurons. Furthermore, PV expression demonstrated the strongest inverse correlation with nitric oxide production.

## 3. Discussion

Hypoxia and ischemia trigger selective neuronal vulnerability in the brain, with GABAergic neurons exhibiting significantly higher sensitivity to damage compared to glutamatergic populations. While pyramidal neurons adapt through hypoxic preconditioning—activating endogenous neuroprotective mechanisms like PI3K/Akt signaling and NMDA/AMPA receptor modulation—GABAergic interneurons fail to mount such defenses effectively. Their heightened metabolic demands, accelerated ROS production, and susceptibility to calcium overload underlie this disparity. Calcium-binding proteins (parvalbumin, calbindin, and calretinin) serve as critical buffers in GABAergic neurons, mitigating excitotoxicity and oxidative stress during hypoxia. However, prolonged ischemia, ultimately, overwhelms these protective systems, leading to irreversible interneuron loss and disrupted network inhibition. This study elucidates the molecular basis of GABAergic neuron vulnerability, focusing on the roles of Ca^2+^ dysregulation, mitochondrial dysfunction, and compromised preconditioning pathways while exploring therapeutic strategies to enhance their survival under ischemic conditions. It should be noted that the neuroprotective effect of calcium-binding proteins (CaBPs) in our study was established only at the correlation level. While the obtained data indicate their protective role, additional experiments on the targeted modulation of PV, CB, and CR expression in GABAergic neurons are required to confirm the causal relationship. It is also promising to study the interaction of CaBPs with other protective mechanisms, such as PI3K/Akt signaling. The obtained results provide a basis for further research into the functional significance of these proteins.

### 3.1. Differential Sensitivity of Neurons to Hypoxia and Ischemia

We have demonstrated that under conditions of both acute and chronic hypoxia GABAergic neurons undergo damage and cell death, whereas glutamatergic neurons adapt to these adverse conditions, exhibiting high survival rates and showing the beneficial effects of hypoxic preconditioning. The oxygen sensor in neurons is mitochondrial and mediates its signaling through reactive oxygen species (ROS), regulating plasma membrane channels. This suggests that differential hypoxia sensitivity of neuronal populations reflects variations in protein activity/expression. GABAergic neurons demonstrate accelerated ROS generation upon AMPA/KA receptor activation, mirroring hypoxia/OGD effects involving glutamate leakage. Notably, this ROS production is calcium-dependent, being abolished in calcium-free medium and restored with physiological Ca^2+^. Glutamatergic neurons show prominent ROS generation at 10 mM extracellular Ca^2+^ [36]. Fast-spiking GABAergic interneurons exhibit particular vulnerability in complexes I/IV of the respiratory chain, consistent with their exceptional energy demands for gamma oscillations [37]. These interneurons show increased mitochondrial density, elevated COX activity, and high cytochrome C expression [38,39].

### 3.2. Hypoxic Preconditioning: Possible Pathways for Preconditioning Activation

Hypoxic preconditioning is an effective way to increase the resistance of organs and tissues to the effects of prolonged hypoxia–reoxygenation and ischemia by applying one [40] or several episodes of short-term hypoxia followed by reoxygenation [41]. It is known that neurons begin to die after ischemia lasting more than 2 min [42]. A reduced oxygen concentration triggers physiological responses such as endothelial proliferation and angiogenesis, which alleviate tissue hypoxia and promote recovery [43]. These positive effects can also occur during hypoxia due to the phenomenon of hypoxic preconditioning. Studies have shown that preconditioning with one short-term (2 min) episode of anoxia in cerebral cortex olfactory zone slices or three 1 min anoxia episodes in hippocampal slices increases cellular resistance to subsequent severe anoxia, preventing action potential suppression and calcium ion overload. Rapid injury and death of the most vulnerable neuronal populations during hypoxia and ischemia trigger glutamate release, which damages neighboring neurons via excitatory receptor activation (primarily NMDA and AMPA) [44]. However, hypoxic episodes also activate preconditioning mechanisms in these receptors, reducing their channel conductivity for Ca^2+^ ions.

However, these properties are characteristic of pyramidal neurons, and our experiments clearly show that, even with low Ca^2+^-signal amplitudes in GAD65/67(+) neurons upon NMDAR activation, episodes of short-term hypoxia consistently increase Ca^2+^-signal amplitudes, indicating a weakening of cellular defense systems. It has been established that hypoxic preconditioning enhances the affinity of GABA receptors for selective agonists [45]. Moreover, preconditioned rat neurons exhibit increased GAD activity, promoting enhanced GABA secretion during subsequent ischemia [46]. In rat cerebral cortex slices, hypoxic preconditioning shifts the excitation–inhibition balance toward GABAergic transmission [47]. However, the brain can maintain this balance and resist hypoxia/ischemia only temporarily; eventually, the most hypoxia-sensitive GABAergic neurons die. Thus, elucidating signaling pathways that protect GABAergic neurons is crucial. Our analysis identified several proteins that enhance preconditioning in glutamatergic neurons and induce this phenomenon in GAD65/67(+) neurons. The PI3K/Akt signaling pathway plays a central role in neuroprotection, synaptic protein trafficking, and protein synthesis control [48]. Its activation induces eNOS activation, NO production, and PKG signaling [49]. These kinases may phosphorylate IP_3_R, thereby suppressing its activity [50]. The NO system and cGMP-dependent kinases also regulate intracellular Ca^2+^ dynamics. L-arginine, which acts via Gi protein-coupled α2-adrenoreceptors, stimulates NO formation and PKG activation in vascular endothelial cells [51]. As an eNOS substrate and α2-adrenoreceptor activator, L-arginine is critical for PKG activation. Previous studies [52] demonstrated that low L-arginine concentrations (0.1–1 mM) in hippocampal neuron cultures stabilize Ca^2+^ responses during excitotoxic glutamate exposure and OGD.

### 3.3. Calcium-Binding Proteins in GABAergic (GAD65/67(+)) Neurons: Correlation with Ischemic Resistance

The results indicate that glutamatergic neurons survive ischemic injury, whereas increased CaBP expression correlates with enhanced viability of GABAergic (GAD65/67(+)) neurons during hypoxia. This is consistent with data reported by other researchers. For example, in ischemia models, primarily striatal interneurons survive, and they typically contain PV as the main CaBP [53]. Middle cerebral artery occlusion (MCAO) in global cerebral ischemia models results in a significant decrease in PV levels in the brain, which ultimately contributes to increased [Ca^2+^]_i_ and neuronal cell death [54]. In amyotrophic lateral sclerosis (ALS), a high correlation has been shown between selective motor neuron damage and the distribution of CaBPs—PV and CB. During ALS development, glutamate toxicity primarily causes death in neurons lacking these CaBPs. By contrast, neurons in the oculomotor nuclei and Onuf’s nucleus, which highly express these proteins, remain undamaged [55]. According to studies [56], the neuroprotective properties of parvalbumin, manifested in the limitation of calcium overload, are negated when the threshold protein concentration in the cell is exceeded. Although our findings demonstrate a correlation between calcium-binding protein levels and neuronal viability, further studies using genetic or pharmacological interventions are required to establish causal mechanisms. The increased (non-physiological) PV content may disrupt normal Ca^2+^ signaling in transfected cells, potentiating their sensitivity to glutamate toxicity. However, transgenic studies show that PV overexpression in spinal cord motor neurons protects them from kainate-induced toxicity [57]. Furthermore, PV-overexpressing mice exhibit altered calcium signaling, including reduced [Ca^2+^]_i_ and enhanced spontaneous transmitter release upon immunoglobulin exposure. Morphologically, PV knockout mice show a 2-fold increase in mitochondrial density in fast-twitch muscles, which may influence Ca^2+^ signaling during contraction [57]. In our in vitro ischemia model, PV was the least-effective CaBP, whereas CR and CB correlate with the strongest neuroprotection. These findings align with retinal neuron studies, where CB was most effective against ischemia/reperfusion injury, while CR offered moderate resistance. In these neurons, PV primarily functions in Ca^2+^-dependent signaling rather than as a buffer protein [58]. We found that PV expression correlates with suppression of ROS production in GAD65/67(+) neuron mitochondria, likely due to its mobility. A study by Henzi et al. [59] found that PV facilitates Ca^2+^ transport to the endoplasmic reticulum and mitochondria, promoting cytoplasmic Ca^2+^ diffusion and signal propagation. Thus, our observed inverse relationship between PV expression and mitochondrial ROS levels (Figure 6B) could theoretically reflect either a direct ROS-scavenging action of PV, as suggested in other systems [57], or secondary effects of improved calcium homeostasis. Without performing PV knockdown/overexpression experiments in our model, we cannot rule out alternative explanations, including coordinated expression of other antioxidant systems.

Thus GABAergic neurons are highly sensitive to hypoxia and ischemia due to their elevated metabolic demands, rapid ROS generation, and susceptibility to calcium overload. In contrast, glutamatergic neurons adapt through hypoxic preconditioning, which reduces excitotoxicity by modulating NMDA/AMPA receptors and limiting Ca^2+^ influx. The PI3K/Akt pathway enhances neuroprotection by activating NO-dependent signaling, while calcium-binding proteins (PV, CB, and CR) mitigate damage by buffering excess Ca^2+^ and reducing oxidative stress. However, prolonged ischemia overwhelms these defenses, leading to selective GABAergic neuron death and network dysfunction. Targeting preconditioning pathways and Ca^2+^ regulation may offer therapeutic strategies for ischemic injury.

This study is primarily descriptive in nature, as we have demonstrated using various in vitro models that specific populations of GABAergic neurons show the lowest tolerance to prolonged hypoxia, hypoxia/reoxygenation episodes, or ischemic-like conditions. Importantly, increased expression of calcium-binding proteins (CaBPs) in GABAergic neurons was correlated with their enhanced resistance to Ca^2+^ overload and cell death. However, it should be emphasized that the observed association does not prove a causal relationship, and further studies employing targeted knockdown or overexpression approaches will be required to elucidate the precise mechanisms underlying the neuroprotective effects of CaBPs.

### 3.4. Limitations of the Study

There are several significant limitations to the work presented. First of all, the data obtained on the protective role of calcium-binding proteins are observational in nature, since the study did not include a targeted genetic intervention to confirm cause-and-effect relationships. The experimental model used with hippocampal cell cultures, despite its informativeness, does not fully reproduce the complex interactions characteristic of the whole organism, in particular the influence of the vascular system and immune factors on neuronal reactions during oxygen starvation. Although this work demonstrates the relationship between the presence of calcium-binding proteins and a decrease in pathological processes, the specific biochemical mechanisms underlying this phenomenon require further study. It should also be taken into account that the pharmacological agents used could have a complex effect that goes beyond the signaling pathways studied. An important aspect that was left out of the study is a differentiated analysis of the various subclasses of GABAergic neurons, which may demonstrate different resistance to damaging effects. In addition, the results obtained with the neonatal cell cultures may not fully reflect the response characteristics of mature human nervous tissue. To overcome these limitations, it seems promising to conduct research using genetic engineering methods, creating more complex experimental systems, as well as a detailed study of the specifics of various neuronal populations.

## 4. Materials and Methods

The experimental protocols were approved by the Bioethics Committee of the Institute of Cell Biophysics. The experiments were carried out according to Act708n (23 August 2010) of the Russian Federation National Ministry of Public Health, which states the rules of laboratory practice for the care and use of laboratory animals, and the Council Directive 2010/63 EU of the European Parliament on the protection of animals used for scientific purposes.

### 4.1. Hippocampal Cell Culture

A mixed-neuroglial cell culture of the hippocampus was obtained from newborn (P1–3) Spraque–Dawley rats. After decapitation, the hippocampus was removed and transferred to a cold Hanks solution. The tissue was minced with scissors, placed in Versene solution with the addition of 0.2% trypsin, and incubated for 10 min at 37 °C on a thermoshaker at 600 rpm. Enzyme-treated tissue pieces were washed three times with neurobasal medium and then carefully pipetted and centrifuged (2 min at 300× *g*). Next, the supernatant was removed, and the cells were resuspended in neurobasal medium supplemented with glutamine (0.5 mM), Supplement B27 (2%), and gentamicin (15 μg/mL). The suspension was added to glass cylinders with ground ends and an internal diameter of 6 mm, standing on round coverslips with a diameter of 25 mm (VWR International); coated with polyethyleneimine; and placed in 35 mm Petri dishes (Greiner). A total of 100 μL of cell suspension was added to each cylinder and left for 2 h for attachment in a CO_2_ incubator at 37 °C. After this, the cylinders were removed, and the volume of the culture medium was adjusted to 1.5 mL. Every 3 days, two-thirds of the volume of the culture medium was replaced with fresh medium [60]. The experiments were carried out on cultures aged 10 days (10 DIV).

### 4.2. Immunocytochemical Method for GABAergic (GAD65/67(+)) Neurons Identifying and Comparing Them with Neuroimaging Results

**Figure 7:**
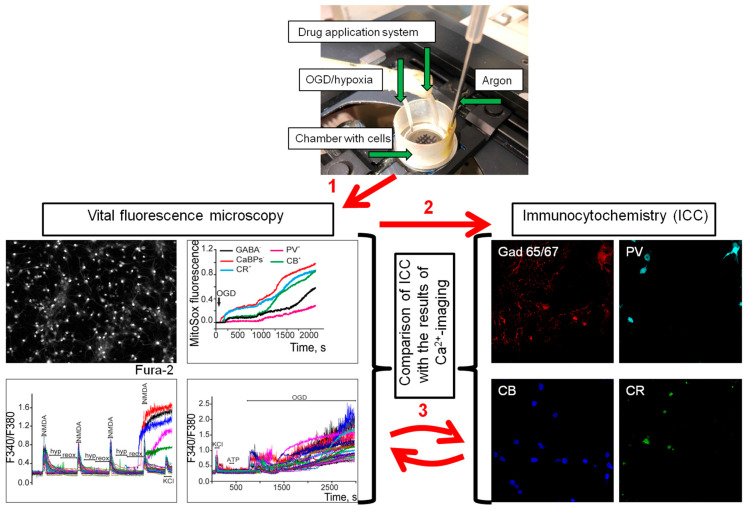
Scheme of the original setup for creating hypoxia/reoxygenation episodes or OGD in vitro and a method for comparing the results of neuroimaging with data from the immunocytochemical staining of cell cultures. Hippocampal neurons were grown on round coverslips and loaded with fluorescent probes. They were mounted in an experimental chamber, and a grid was applied to the bottom of the glass with a waterproof marker. The chamber with cells was mounted on the stage of a fluorescence microscope, equipment for working with hypoxia was brought in, and an experiment was conducted to record the vital physiology of neurons. Then, the cells were fixed and loaded with antibodies. The chamber with cells was transferred to a confocal microscope, the area of interest was found using a grid, in which the intravital parameters of cells under hypoxic conditions were previously recorded, and fluorescence imaging of secondary antibodies was performed. The confocal images were then compared with lifetime neuroimaging data to identify signal patterns characteristic of certain subtypes of GABAergic (GAD65/67(+)) neurons. Red numbers 1–3 indicate the following sequence of manipulations: 1—registration of Ca^2+^ dynamics, ROS production, or mitochondrial potential during hypoxia or OGD; 2—fixation of cells and staining with antibodies against GAD65/67, parvalbumin, calbindin, and calretinin, with secondary antibody fluorescence registration performed using a confocal microscope; and 3—comparison of cell images obtained in stage 1 and stage 2 and the identification of vital fluorescent signals characteristic of a certain type of neuron.

An immunocytochemical method was used to identify GABAergic neurons containing CaBPs (Figure 7). The cover glass with the culture was mounted in the experimental chamber and a grid was applied on the bottom side of the glass using a thin indelible marker for further detection of the area of interest with cells on different microscopes and comparison of vital imaging and culture data after staining with antibodies. Next, the chamber was mounted on the stage of an inverted microscope, one of the squares of the drawn grid was selected, and the fluorescence was recorded in the selected area. After the experiments, the cells were photographed in phase contrast, numbered, fixed, and analyzed by immunocytochemically staining with antibodies. We performed parallel staining of neurons with rabbit polyclonal antibodies against glutamate decarboxylase (GAD65/67, ab 11070) and calcium-binding proteins, sheep polyclonal anti-parvalbumin antibodies (Parvalbumin antibody, ThermoFisher, Waltham, MA, USA, PA5-47693), goat polyclonal anti-calbindin antibodies (Calbindin D28K antibody, SantaCrus, Mumbai, India, sc-7692), and mouse monoclonal anti-calretinin antibodies (Calretinin antibody [6B3], Abcam, Cambridge, UK, ab82198). For this purpose, the following solutions were used: phosphate-buffered saline (PBS), pH 7.4; 4% parapharmaldehyde solution in PBS; and 1% and 10% solutions of donkey serum in PBS. Cells were fixed in 4% parapharmamaldehyde for 20 min and then washed three times for 5 min with cold PBS and permeabilized in 0.1% Triton X-100 in PBS for 15 min. To inhibit sites’ non-specific antibody binding, cells were incubated for 30 min in 10% donkey serum at room temperature and then incubated with secondary antibodies of Donkey Anti-Rabbit IgG H&L Alexa Fluor^®^ 555 (Abcam, ab150074), Donkey Anti-Sheep IgGH&L Alexa Fluor^®^ 647 (Abcam, ab150179), Donkey Anti-Goat IgG H&L Alexa Fluor^®^ 488 (Abcam, ab150129), and Donkey Anti-Mouse IgG H&L Alexa Fluor^®^ 594 (Abcam, ab150108) for 12 h at 4 °C (1:500 dilution in 1% donkey serum); washed in PBS 3 times for 5 min; and incubated with secondary antibodies conjugated to a fluorescent tag. Antibody fluorescence was recorded on a Leica TCS SP5 confocal microscope (Leica, Wetzlar, Germany) equipped with a He-Ne laser. Images of antibody fluorescence and intravital imaging were compared using ImageJ software, v 1.54d (developed by LOCI at the University of Wisconsin, Madison, WI, USA, available at https://imagej.nih.gov/ij/download.html, accessed on 18 May 2023, RRID: SCR_003070). This approach allowed us to compare changes in the [Ca^2+^]_i_ dynamics, ROS and nitric oxide production in GAD 65/67-positive (GAD65/67(+)) neurons containing calcium-binding proteins [61,62].

### 4.3. Fluorescence Measurements

Cells were stained with the probe Fura-2AM (4 µM; 40 min incubation; 37 °C) dissolved in Hank’s balanced salt solution (HBSS) consisting of (mM) 156 NaCl, 3 KCl, 2 MgSO_4_, 1.25 KH_2_PO_4_, 2 CaCl_2_, 10 glucose, and 10 HEPES (pH 7.4) and then washed out for 15 min. To record the level of calcium in the cytoplasm of cells ([Ca^2+^]_i_), we used a Cell Observer image analysis system (Carl Zeiss, Jena, Germany) based on an Axiovert 200M inverted microscope equipped with an AxioCam HSm monochrome CCD camera and a Ludl MAC5000 high-speed excitation filter changing system. A Plan-Neofluar 10×/0.3 lens was used. An HBO 103W/2 mercury lamp illuminator was used as a fluorescence excitation source. To excite and record Fura-2 fluorescence, we used the 21HE filter set (Carl Zeiss, Germany) with the BP340/30 and BP387/15 excitation filters, an FT409 beam splitter, and an BP510/90 emission filter. To measure fluorescence, a round cover glass with a cell culture was mounted in a special measuring chamber. The volume of medium in the chamber was 0.5 mL. Reagents were added and washed by replacing the medium tenfold using a system providing perfusion at a rate of 15 mL/min. Measurements were carried out at 28 °C. A series of images was acquired at intervals of 1 frame every 3 s. To identify neurons, the experiment included a short-term (30 s) test application of 35 mM KCl. The KCl-induced depolarization promotes the opening of voltage-gated Ca^2+^ channels and induces rapid Ca^2+^ responses exclusively in neurons. The conductivity and density of cation channels in astrocytes are insufficient to evoke a high-amplitude Ca^2+^ response to KCl. But the application of 10 µM ATP induced activation of purinoreceptors and Ca^2+^ responses exclusively in astrocytes [23,24]. The resulting time series of two-channel images (at excitation light wavelengths of 340 and 380 nm) were processed in the ImageJ software with the Time series analyzer software module. The amplitude of the calcium responses of single cells was measured and expressed as the ratio of Fura-2 fluorescence signals at excitations of 340 and 380 nm. Origin 8.5 was used for plotting and statistical processing. The results are presented as single-cell signals, generally representative of the GABAergic neuron subtype shown or as the average cell signal per the field of view ± standard deviation (SD).

Nitric oxide (NO) production was measured using the fluorescent probe DAF-FM diacetate (Molecular Probes, Eugene, OR, USA). Cells were loaded with 5 μM DAF-FM for 40 min at 37 °C. After loading, the cells were additionally incubated in Hanks balanced salt solution (HBSS) for 20 min to complete the de-esterification of the dyes. DAF-FM fluorescence was excited using a BP 475/40 filter (Carl Zeiss, Jena, Germany). The emission of DAF-FM was recorded at 530 ± 25 nm [63,64]. The collected 8-bit time-lapse images were analyzed using ImageJ software with the Time Series Analyzer and RatioPlus plugins. The experimentally obtained curves for NO were smoothed to decrease the effect of noises. The curve ΔF/F0 characterizes the fluorescence intensity of benzotriazole, a product of the nitrosylation of the DAF-FM accumulated in the cells.

For simultaneous recordings of the changes in [Ca^2+^]_i_ and mitochondrial or cytosolic ROS production, hippocampal cell cultures were loaded with Fura-2AM (4 µM; 40 min incubation; 37 °C) and MitoSOX Red (mitochondrial ROS indicator; 5 µM; 15 min incubation; 37 °C) or H_2_DCF-DA (mainly cytosolic ROS indicator; 10 µM; 20 min incubation; 37 °C). Cells were stained with the probes dissolved in Hank’s balanced salt solution (HBSS), composed of (mM) 156 NaCl, 3 KCl, 2 MgSO_4_, 1.25 KH_2_PO_4_, 2 CaCl_2_, 10 glucose, and 10 HEPES, pH 7.4. After incubation with the dyes, cells were washed three times before the experiment. To measure the cytosolic free Ca^2+^ concentration ([Ca^2+^]_i_) and ROS generation, we used a system based on a Leica DMI6000B inverted motorized microscope with a high-speed monochrome CCD-camera (HAMAMATSU C9100) and a high-speed light filter replacing system (Leica’s Ultra-Fast Filter Wheels) with replacing time of 10–30 ms. For the excitation and registration of the Fura-2 fluorescence, we used the FU-2 filter set (Leica, Germany) with the BP340/30 and BP387/15 excitation filters, an FT-410 beam splitter, a BP510/84 emission filter, objective Leica HC PL APO 20./0.7 IMM, and Leica EL6000 excitation light source, with an HBO 103 W/2 high-pressure mercury lamp. For excitation of the DCFH_2_-DA and MytoSOX Red, we used the L5 filter set (Leica, Germany) with the BP480/40 excitation filter, 505 dichroic mirror, and 527/30 emission filter [65]. In order to prevent non-specific photooxidation of the ROS indicators, the laser power was decreased to 3–5%. It should be noted that each probe was excited alternately, which allowed us to divide the fluorescent signals of each probe and exclude non-specific emission collection. The shape and speed of the ROS production rates under oxygen–glucose deprivation (OGD) were determined. The ImageJ, Origin 8.5, and Prism GraphPad software (GraphPad Software, RRID: SCR_002798, V 8.0.1) applications were used in order to analyze the data, create graphs, and perform statistical tests. All values are given as the mean ± SEM. All presented data were obtained from at least three cover slips and two to three different cell preparations.

### 4.4. Technique for the Simulation of Ischemia-like Conditions

Ischemia-like conditions (oxygen–glucose deprivation, OGD) were obtained by omitting glucose (HBSS medium without glucose) and by displacing dissolved oxygen with nitrogen or argon in a special leak-proof system. The level of oxygen in the medium was measured using a Clark electrode. Oxygen tensions reached values of 30–40 mm Hg or less within 20 min after the start of displacement. Ischemia-like conditions lasting for 40 min were created by means of supplying the oxygen–glucose deprivation (OGD) medium into the chamber, which contained cultured hippocampal cells. A constant argon feed into the experimental chamber was used to prevent contact between the OGD medium and the atmospheric air. The effects of OGD on neurons and astrocytes were evaluated by measuring the amplitude of the cell calcium response and the shape and rate of ROS production, as well as assessment of the cell viability before and after the ischemia-like conditions.

### 4.5. Technique for the Modeling of Short-Term Hypoxia Episodes and Long-Term Hypoxia

To model hypoxic conditions, we used an HBSS solution with a low concentration of dissolved oxygen. HBSS was purged for 15 min with argon in a special hermetic system to displace oxygen. Oxygen tension was measured with a Clark electrode and reached 50–60 mm Hg, which corresponds to moderate hypoxia. Each hypoxia/reoxygenation cycle consisted of a 3 min episode of hypoxia when the hypoxic medium was added to the experimental chamber with the hippocampal cell culture and a 10 min reoxygenation episode when the cultures were washed with the HBSS solution with a normal concentration of dissolved oxygen. Short-term applications (30 s) of NMDA receptor agonists were conducted after each hypoxia/reoxygenation cycle. The drugs were added with a flow rate of 10 mL/min using the designed perfusion system. The volume of the medium in the experimental chamber was 500 µL, while the excess liquid was drained with a water-jet pump. Inlet and outlet pipes were installed in front of each other in the experimental chamber. Using a colored solution, we determined that the complete replacement of the bathing solution in the chamber was observed 20–30 s after the start of perfusion. This system allows for performing short-term and long-term applications of the drugs. It was established in control experiments that the responses of the cells to the mechanical stimulation, caused by the flow of the liquid during the application of drugs, are negligible or even absent.

The development of the preconditioning effect was estimated by the change in the amplitudes of the NMDA-induced Ca^2+^ responses after the hypoxia/reoxygenation cycles. The amplitudes of the calcium responses to NMDA application before and after three episodes of hypoxia/reoxygenation were calculated for all analyzed neurons. The calculated values are presented in a coordinate plane. The values of the amplitudes before hypoxia were arranged along the X-axis, while the values of the post-hypoxic amplitudes were arranged along the Y-axis. Then the data points were approximated by linear regression. The slope of the regression lines was used to estimate the preconditioning effect of the hypoxia episodes. A decrease in the slope indicated the development of preconditioning [66].

### 4.6. Assessment of Cell Viability

Cell death induced by OGD or hypoxia exposure was assessed by propidium iodide (PI, 1 µM) before and after OGD/hypoxia in the same microscopic field. Since PI stains both dead astrocytes and neurons, an analysis of the calcium signals upon 35 mM KCl application before OGD/hypoxia was used to identify the type of cell. Neurons were identified by the quick transient calcium signal upon KCl addition, as described previously. Furthermore, we used the Ca^2+^ signals (presence or absence of a global increase in [Ca^2+^]_i_ during OGD/hypoxia) as an additional indicator of cell viability.

### 4.7. Image Analysis and Statistical Data Processing

The resulting time series of images was processed by ImageJ with the Time Series Analyzer plugin. In this case, the amplitude of signals from single cells was measured and expressed in units of probe fluorescence. The results are presented as single-cell signals or the average cell signal per the field of view ± standard error (SE). Origin 8.5 (Microcal Software Inc., Northampton, MA, USA) and Prism 5 (GraphPad Software, La Jolla, CA, USA) were used for plotting and statistical processing. We used the Shapiro–Wilk test to determine the normality of the distribution. Statistical analyses were performed by the Student’s *t*-test. Differences are significant at * *p* < 0.05, ** *p* < 0.01, *** *p* < 0.001, and **** *p* < 0.0001; n/s—data not significant (*p* > 0.05).

## 5. Conclusions

The differential sensitivity of hippocampal neurons to hypoxia, hypoxia/reoxygenation episodes, and ischemia-like conditions was established. GABAergic (GAD65/67(+)) neurons are the most vulnerable type of neurons to such conditions due to the absence of an endogenous protective mechanism of hypoxic preconditioning and die due to the induction of a global [Ca^2+^]_i_ increase. However, the expression of calcium-binding proteins in the cytosol of GAD65/67(+) neurons represents an alternative pathway for their resistance to hypoxia and ischemia, since high levels of calbindin or calretinin expression correlate with a suppression of the global [Ca^2+^]_i_ increase, while parvalbumin expression correlates with a marked suppression of free radical production.

## Figures and Tables

**Table 1:** The effects of activators/inhibitors of key intracellular kinases and associated signaling cascades on the mechanism of hypoxic preconditioning in glutamatergic (GAD65/67(−)) and GAD65/67(+) neurons of the hippocampus. The tangent of the slope of the straight line, approximating the amplitudes of neuronal Ca^2+^ responses to the application of NMDA after 3 episodes of hypoxia, was analyzed. The number of dead cells after the experiments was assessed by staining the hippocampal cultures with propidium iodide. Statistical analyses were performed using the Student’s *t*-test. Comparisons were made relative to the control within the group of GAD65/67(−) neurons (black asterisks) and separately within the group of GAD65/67(+) neurons (red asterisks). Differences are significant at *** *p* < 0.001, ** *p* < 0.01 and * *p* < 0.05; n/s—differences are not significant (*p* > 0.05). For each panel, the experiments were performed in 6 replicates. Slope coefficients reflect changes in [Ca^2+^]_i_ in response to NMDA; cell death was assessed independently using PI.

Drug, Concentration (µM) and Intracellular Target	Slope Coefficient of Linear Approximation After 3 Episodes of Hypoxia		% of Dead Neurons
	GAD65/67(−)	GAD65/67(+)	
Control	0.47 ± 0.07	1.12 ± 0.05	3 ± 1%
LY-294,002 (1 μM), PI3K (inhibitor)	0.75 ± 0.11 **	1.2 ± 0.18 *	6 ± 2% ***
LY-294,002 (10 μM), PI3K (inhibitor)	0.87 ± 0.05 ***	1.4 ± 0.06 **	8 ± 1% ***
Wortmannin (1 μM), PI3K (inhibitor)	0.67 ± 0.1 *	1.34 ± 0.16 **	7 ± 1% **
Wortmannin (5 μM), PI3K (inhibitor)	0.85 ± 0.06 ***	1.34 ± 0.16 **	4 ± 1% ^n/s^
740 Y-P (1 μM), PI3K (activator)	0.36 ± 0.06 *	0.63 ± 0.04 ***	1 ± 1% *
7NI (5 μM), NOS (inhibitor)	0.46 ± 0.07 ^n/s^	0.56 ± 0.21 ***	5 ± 3% ^n/s^
SNAP (5 μM), NOS (activator)	0.77 ± 0.06 **	1.59 ± 0.11 ***	16 ± 3% ***
L-arginine (5 μM), NOS, and α2-adrenoreceptor (activator)	0.48 ± 0.07 ^n/s^	0.54 ± 0.2 ***	4 ± 2% ^n/s^
Rp-8-pCPT-cGMPS (1 μM), PKG (inhibitor)	0.73 ± 0.03 **	1.24 ± 0.09 **	11 ± 2% ***
8-Br-cGMP (10 μM), PKG (activator)	0.49 ± 0.06 ^n/s^	0.8 ± 0.07 ***	3 ± 2% ^n/s^

## Data Availability

The data presented in this study are available upon request from the corresponding author.

## Supplementary Materials

The following supporting information can be downloaded at: https://www.mdpi.com/article/10.3390/ijms26167966/s1.
