# Supplementary material for: Differential Sensitivity of Hippocampal GABAergic Neurons to Hypoxia and Ischemia-like Conditions Correlates with the Type of Calcium-Binding Protein Expressed
Source: Int J Mol Sci. 2025 Aug 18;26(16):7966. doi: 10.3390/ijms26167966 (PMC12386940; doi:10.3390/ijms26167966)
Supplement: Supplementary file 1 [file ijms-26-07966-s001.zip › ijms-3681022-supplementary.pdf]

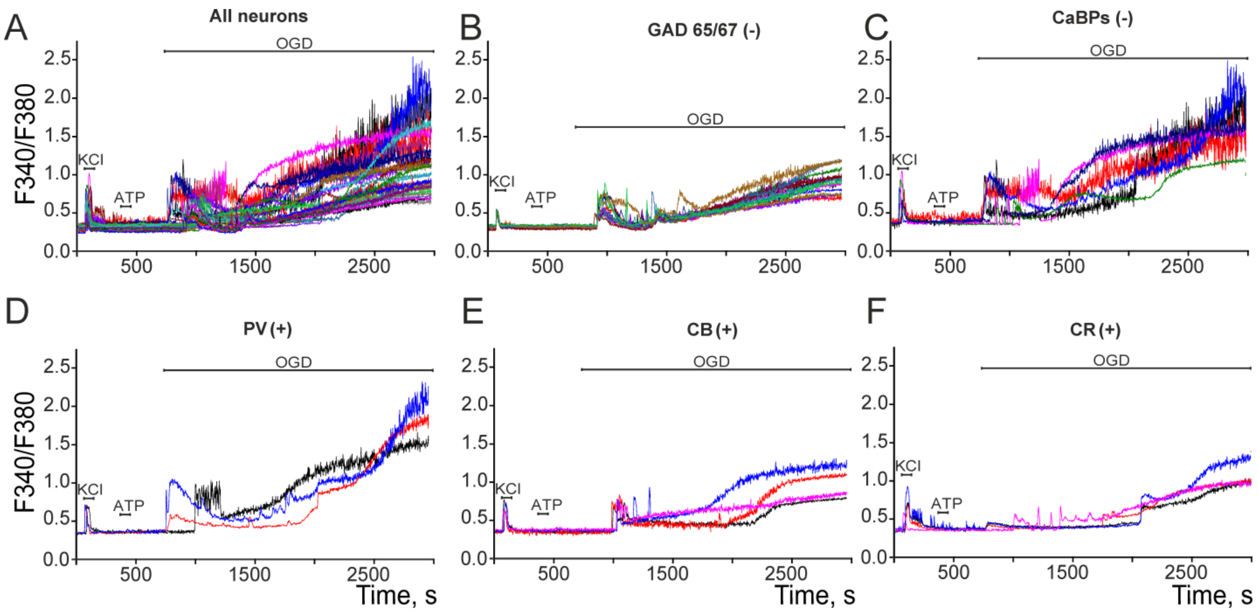

Figure S1. Ca^{2+} signals in different types of hippocampal neurons on ischemia-like conditions (OGD, ~40 min). The role of calcium-binding proteins expression in GABAergic neurons. **A** – Ca^{2+} signals of all hippocampal neurons in the field of view of the microscope on ischemia-like conditions (OGD), applications of 35 mM KCl and ATP (10 μM). **B**, **C**, **D**, **E**, **F** – Ca^{2+} signals on OGD in GABA neurons (**B**), GABAergic neurons without expression of the studied calcium-binding proteins (**C**, $CaBPs^{-}$), expressing parvalbumin (**D**, PV^{+}), calbindin (**E**, CB^{+}) or calretinoin (**F**, CR^{+}).
